# Experiences with and expectations of maternity waiting homes in Luapula Province, Zambia: a mixed–methods, cross-sectional study with women, community groups and stakeholders

**DOI:** 10.1186/s12884-017-1649-1

**Published:** 2018-01-25

**Authors:** Peggy S. Chibuye, Eva S. Bazant, Michelle Wallon, Namratha Rao, Timothee Fruhauf

**Affiliations:** 1 8 Ngumbo Road, Longacres, 10101 Lusaka, Zambia; 2 Jhpiego, an affiliate of Johns Hopkins University, 1615 Thames Street, Baltimore, MD 21231-3492 USA; 3 Walko Global Development Partners, LLC, 805 Sycamore Place, Ann Arbor, MI 48104 USA; 4 Johns Hopkins Bloomberg School of Public Health, 615 N. Wolfe Street, Baltimore, MD 21205-2103 USA

**Keywords:** Maternity waiting home, Maternal health, Infrastructure, Safe motherhood action groups, Luapula, Zambia

## Abstract

**Background:**

Luapula Province has the highest maternal mortality and one of the lowest facility-based births in Zambia. The distance to facilities limits facility-based births for women in rural areas. In 2013, the government incorporated maternity homes into the health system at the community level to increase facility-based births and reduce maternal mortality. To examine the experiences with maternity homes, formative research was undertaken in four districts of Luapula Province to assess women’s and community’s needs, use patterns, collaboration between maternity homes, facilities and communities, and promising practices and models in Central and Lusaka Provinces.

**Methods:**

A cross-sectional, mixed-methods design was used. In Luapula Province, qualitative data were collected through 21 focus group discussions with 210 pregnant women, mothers, elderly women, and Safe Motherhood Action Groups (SMAGs) and 79 interviews with health workers, traditional leaders, couples and partner agency staff. Health facility assessment tools, service abstraction forms and registers from 17 facilities supplied quantitative data. Additional qualitative data were collected from 26 SMAGs and 10 health workers in Central and Lusaka Provinces to contextualise findings. Qualitative transcripts were analysed thematically using Atlas-ti. Quantitative data were analysed descriptively using Stata.

**Results:**

Women who used maternity homes recognized the advantages of facility-based births. However, women and community groups requested better infrastructure, services, food, security, privacy, and transportation. SMAGs led the construction of maternity homes and advocated the benefits to women and communities in collaboration with health workers, but management responsibilities of the homes remained unassigned to SMAGs or staff. Community norms often influenced women’s decisions to use maternity homes. Successful maternity homes in Central Province also relied on SMAGs for financial support, but the sustainability of these models was not certain.

**Conclusions:**

Women and communities in the selected facilities accept and value maternity homes. However, interventions are needed to address women’s needs for better infrastructure, services, food, security, privacy and transportation. Strengthening relationships between the managers of the homes and their communities can serve as the foundation to meet the needs and expectations of pregnant women. Particular attention should be paid to ensuring that maternity homes meet quality standards and remain sustainable.

**Electronic supplementary material:**

The online version of this article (10.1186/s12884-017-1649-1) contains supplementary material, which is available to authorized users.

## Background

Zambia has recently made significant progress in reducing maternal mortality. The maternal mortality ratio declined from 729 in 2001 [1] to 591 in 2007 [2] to 398 per 100,000 live births between 2013 and 2014 [3]. Yet, these figures remain among the highest in the region and have been linked to the place of childbirth among other factors. According to the 2013–2014 Demographic and Health Survey, 67.4% of pregnant women in Zambia delivered in a facility in the preceding 5 years [3], a number limited by distance to health facilities [3, 4]. A 2011 study linking household and facility data through a Geographic Information System in Zambia found that half of rural births are among mothers living 25 km or more from a health facility offering adequate maternal health care services; the likelihood of giving birth in a facility decreased by 29% when distance doubled [4]. While women cite many reasons for home deliveries, most women (31.9%) noted that distance to facilities and the absence of transportation were the main reason [3].

In an effort to increase deliveries in health facilities, the Zambian government integrated maternity waiting homes into the health system at the level of Community Rural Health Centres (CRHCs) in its 2013 Roadmap for Accelerating Reduction of Maternal, Newborn, and Child Mortality [5]. Maternity waiting homes are residential structures where pregnant women can wait for delivery during the final weeks of their pregnancy [6]. They are usually located near a hospital that provides essential obstetric care [6, 7] or a health centre that can refer women with complications to the hospital [7]. The World Health Organisation has endorsed them as one component of a comprehensive package to reduce maternal morbidity and mortality [4].

Since their integration into the Zambian health system in 2013, maternity waiting homes, often referred to as maternity homes [8], have become formally linked to facilities. Prior to that, the homes had been tied to faith-based facilities or community groups. While only two faith-based facilities and one government facility had maternity homes in 2011, that number has since grown rapidly.

The effectiveness of maternity homes in increasing access to skilled birth attendants and facility-based deliveries has been described in many resource-poor settings across sub-Saharan Africa [9–14]. While the reduction in time needed to respond to obstetric complications has not been quantified, several studies have reported positive effects on maternal mortality, pregnancy complications, and neonatal outcomes [10, 15, 16]. In the Eastern province of Zambia, identical maternal health outcomes in women who used and did not use maternity waiting homes were reported despite the higher risk profiles of women staying in maternity homes in 1994 [17].

However, in many settings, pregnant women did not always use maternity homes, thereby limiting their impact on health [17–19]. In Kalabo District in the Western Province of Zambia, women articulated the benefits of maternity homes and expressed a desire to utilise them, but their lack of decision-making autonomy, concerns about child care at home, inability to purchase required goods, and inadequate services at maternity homes discouraged their use of the homes [20]. Facilitators and barriers have affected the utilization of maternity homes in several countries [8–20].

The barriers and facilitators of utilization of maternity homes are rarely investigated in the context of an evaluation. Furthermore, most studies have left out key stakeholders from assessments thereby incompletely describing the interplay between pregnant women, communities, maternity homes, and facilities that significantly affect use of the homes. Finally, factors affecting the homes’ use have been found to be context-specific. For example, traditional midwives encouraged more women to use maternity homes in Liberia [10] but traditional birth attendants were barriers to maternity home use in Ghana where women were not comfortable with the volunteers [21]. In the context of Zambia’s recent investment in maternity home construction, it is critical to further explore the factors that affect use in communities that now have access to maternity homes.

This study grew out of an evaluation of maternity homes requested by Merck for Mothers, an international grant-making organisation dedicated to improving access to maternal care in resource-limited settings and committed to evaluating the effectiveness of maternal health interventions. This research took place in Mansa, Chembe, Samfya and Lunga Districts of Luapula Province in Zambia. This manuscript focuses on understanding the experiences with and expectations of maternity home users, community groups and other stakeholders to identify facilitators and barriers of use, and determine how these expectations shape the current use and the sustainability of maternity homes. More particularly, this research seeks to answer three questions:

1. To what extent do the infrastructure and amenities at maternity homes at mission hospitals and CRHCs meet the community’s and women’s expectations?
2. How do these expectations affect the current use of maternity homes at mission hospitals and CRHCs?
3. What roles do community groups and health facility staff play in establishing, operating, and advocating for the sustainable use of maternity homes in mission hospitals and CRHCs?

## Methods

### Setting

This study focuses on Luapula Province in northern Zambia. Luapula Province is a sparsely populated (19.6 persons per square kilometre), primarily rural province with poor roads and expansive swamps [22]. It has the highest maternal mortality ratio in Zambia at 573 per 100,000 live births, compared to 483 per 100,000 live births nationally [22]. In 2013–2014, 68.4% of live births occurred in health facilities in Luapula Province [3]. The study also draws lessons from successful maternity homes in the Central and Lusaka Provinces. All study districts are similar in terms of population, road access, livelihood, [22] and poverty level [23].

At the time of this study, the health system comprised 68 health facilities, 21 of which had maternity homes in Luapula Province. There were 204 health facilities in Central Province and 294 health facilities in Lusaka Province [24]. In addition, in rural areas community groups, including chiefs or village headmen, Safe Motherhood Action Groups (SMAGs), Neighbourhood Health Committees, and traditional birth attendants promote reproductive, maternal, neonatal and child health (RMNCH), and HIV services [5].

### Study design

This study employed a mixed–methods, cross-sectional research design [25, 26]. Data were collected between September and December 2013. Three data extraction tools described below were used to collect quantitative data. Qualitative data were collected through focus group discussions and key informant interviews in the four districts in Luapula Province. Additional qualitative data were collected in Serenje and Mkushi Districts in Central Province and Rufunsa District in Lusaka Province to contextualize findings from Luapula Province.

### Sampling of sites and participants

Luapula Province was selected to be the focus of this study by the Ministry of Health, Provincial and District Medical Office Managers because it reported the highest maternal mortality ratio in Zambia [22]. In Luapula Province, only these four districts had maternity homes in 2013 [24]. The three districts in Central and Lusaka Provinces were selected because they had well-integrated and functioning maternity homes. Purposeful sampling was used to select the sites and recruit focus group discussion and key informant interview participants in all provinces [26].

In Luapula Province, qualitative data were collected from 17 of 21 facilities with maternity homes in 2013. The four maternity homes not included were incomplete at the time of data collection. In Central and Lusaka Provinces, qualitative data were collected from three CRHCs and a mission hospital that had maternity homes [26].

In the four districts of Luapula Province, 21 focus group discussions were conducted with 210 participants who attended antenatal care, postnatal care, family planning and children’s clinics in CRHCs and mission hospitals and members of the SMAGs. The participants were maternity home users, non-maternity home users, women who were pregnant for the first time, women who had delivered at home in 2012, elderly women and SMAG/Neighbourhood Health Committees members (Table 1). Each focus group discussion included eight to 12 participants. Interviews were conducted with 21 health facility in-charges, 10 couples from antenatal care clinics, 17 chiefs (four chiefs were unable to participate due to unforeseen circumstances), 12 village headmen, four District Community Health Officers, two District Community Nursing Officers and three staff members from partner agencies (Table 2).

**Table 1:** Number of focus group discussions and participants in Luapula, Central and Lusaka Provinces

Type of Participant	Number of focus group discussions [participants]
*Past users of maternity homes:* women who delivered in a facility within the past year and currently pregnant women	3 [30 participants]
*Non-maternity home users*: women who delivered in a facility within the past year and currently pregnant women	4 [40 participants]
*Women who have not yet delivered in a facility*: women who gave birth at home or with a traditional birth attendant within the past year, or women who are currently pregnant for the first time	4 [40 participants]
S*afe Motherhood Action Grou*ps and Neighbourhood Health Committees	10 [86 participants]
Senior Women	4 [40 participants)
Total	25 [236 participants]

**Table 2:** Number of key informant interview participants in Luapula, Central and Lusaka Provinces

Position	Number of participants
Health facility in-charges	26
Couples: women with spouses at their first antenatal care visit	20
Traditional leaders (Chiefs and Village Headmen)	29
District Community Medical Officers/District Community Nursing Officers	9
Partners agency staff supporting Reproductive, Maternal, Neonatal, and Child Health	3
Total	87

In the Central Province districts, four focus group discussions with six to 12 participants were conducted with 26 SMAG members responsible for mobilizing communities and organizing activities to support maternity waiting homes [26]. Rufunsa District had no SMAGs. In the three districts of Central and Lusaka Provinces, interviews were conducted with three District Community Medical officers, two District Nursing Officers and five health facility in-charges who supervised health staff and had developed systems to work with community groups on maternity homes [26].

### Data collection

Field guides were used by interviewers to guide focus group discussions and key informant interviews (Additional file 1). Focus group discussions focused on maternity home use, food availability, customs and traditions related to facility-based deliveries, maternity homes’ cost and length of stay, willingness to pay, transportation services for pregnant women, and general impressions of maternity homes. Key informant interviews investigated support for maternity waiting homes, mechanisms to sustain maternity homes and their operations. Focus group discussions and interviews lasted approximately 30 to 45, and 20 to 30 min, respectively.

In Luapula Province, quantitative data was collected using three assessment tools. The Maternity Home Assessment Tool containing 42 items was used to collect data through direct observation and clinic staff interviews about the structures and amenities available in maternity homes (Additional file 2). In particular, this tool was created by the research team to collect data on maternity home ownership, funding, and building materials, as well as availability of water and electricity, rooms, beds, mattresses and cooking amenities. A Service Abstraction Form containing 23 items was used to extract annual deliveries from maternity registers accessed through facility in-charges (Additional file 3). A separate assessment tool created by Integrated Rural Development Initiative and Jhpiego was used to collect water sources and sanitation data.

Nine trained research assistants collected all data in Mansa, Chembe, Samfya and Lunga districts. They received training on research ethics, the study’s protocol and data collection tools, and empirical content on collection and quality of qualitative and quantitative data through didactic and hands-on sessions. The first author supervised this team and collected data in Serenje, Mkushi and Rufunsa districts. Focus group discussions and key informant interviews were conducted in the local language, Bemba, until saturation was reached [27]. The data collection tools were piloted with 20 midwives at Levy Mwanawasa General Hospital and Bauleni, Chilenje and Kabwata Health Centres in Lusaka Province and revised before the study started.

### Data analysis

Qualitative data were transcribed from audio recordings in Bemba, translated into English, and back-translated into Bemba to ensure accuracy of the translation. The data were coded in Atlas-ti using codes derived from the field guide questions and emergent themes [28]. Working matrices were used to organize passages and themes by participant type and analysed each district’s responses in a framework [27]. The data coders, three based at Jhpiego in Baltimore and three based in Lusaka, refined the themes, found commonalities, wrote up findings, and returned iteratively to the raw data to find relationships between themes. Quantitative variables were entered into a Microsoft Access database and analysed with descriptive statistics by district using Stata [29]. Results were shared with stakeholders at a dissemination meeting in Lusaka.

### Ethics

The study was approved by the Johns Hopkins University Institutional Review Board in Baltimore and the University of Zambia Research Ethics Committee. All focus group and interview participants were consented verbally prior to their participation in the study. A waiver of written consent was obtained as the study posed no more than minimal risk of harm.

## Results

### Infrastructure and amenities

All the maternity homes had modern structures built with cement brick walls and steel roofs (Table 3). The number of rooms and available supplies differed between maternity homes. Mission hospital homes had an average 2.5 rooms and access to beds, mattresses and linens. Meanwhile, CRHC maternity homes had an average of 1.1 rooms and only 64%, 36% and 15% provided beds, mattresses and linen to women, respectively. These differences mattered to previous and potential maternity home users:

*Women do not use maternity homes because there are no blankets […] some pregnant women cannot manage to carry blankets from home (Woman who gave birth in a CRHC).*

**Table 3:** Maternity home characteristics by district and facility type

	District (Facility type)			
	Mansa (CRHCs)	Chembe (CRHCs)	Samfya (CRHCs)	Samfya (MH)
	*N* = 10	*N* = 1	*N* = 4	*N* = 2
*Structural Characteristics, Continuous: mean (median)*				
Number of rooms	1.1 (1.0)	1.0 (0.0)	1.0 (1.0)	2.5 (2.5)
Number of postpartum beds	1.1 (2.3)	2.0 (0.0)	1.0 (1.7)	14.0 (1.4)
Number of toilet facilities	1.4 (1.0)	0 (0.0)	0.33 (0.0)	2.5 (2.5)
*Structural Characteristics, Categorical: n (%)*				
Flooring material: cement	10 (100.0)	1 (100.0)	4 (100.0)	2 (100.0)
Roofing material				
Metal/iron sheets Calamine/cement fiber	9 (90.0) 1 (10.0)	1 (100) 0 (0.0)	4 (100) 0 (0.0)	2 (100.0) 0 (0.0)
Wall material				
Cement blocks Bricks Cement	7 (70.0) 0 (0.0) 3 (30.0)	0 (0.0) 0 (0.0) 1 (100.0)	0 (0.0) 1 (100.0) 0 (0.0)	0 (0.0) 1 (50.0) 1 (50.0)
Electricity source				
National grid (ZESCO) None	9 (90.0) 1 (10.0)	1 (100.0) 0 (0.0)	1 (25.0) 3 (75.0)	2 (100.0) 0 (0.0)
Electricity present at assessment visit	8 (80.0)	1 (100.0)	0 (0.0)	2 (100.0)
*Goods/Services available, n (%)*				
Mattresses	7 (70.0)	1 (100.0)	2 (50.0)	2 (100.0)
Beds	3 (30.0)	1 (100.0)	2 (50.0)	2 (100.0)
Linens	2 (20.0)	0 (0.0)	0 (0.0)	2 (100.0)
Cooking pots	3 (30.0)	0 (0.0)	2 (50.0)	0 (0.0)
Plates	3 (30.0)	0 (0.0)	2 (50.0)	0 (0.0)
Spoons	1 (10.0)	0 (0.0)	0 (0.0)	2 (100.0)
Cooking utensils	4 (40.0)	0 (0.0)	2 (50.0)	0 (0.0)
Bed nets	1 (10.0)	0 (0.0)	0 (0.0)	2 (100.0)
Telephones	0 (0.0)	0 (0.0)	0 (0.0)	0 (0.0)
*Water and Sanitation, n (%)*				
Main water source				
Protected borehole Piped water	9 (90.0) 1 (10.0)	0 (0.0) 1 (100.0)	2 (50.0) 1 (25.0)	0 (0.0) 2 (100.0)
Type of toilet facilities				
Flush/pour flush to piped sewer system Flush/pour flush to septic tank Ventilated improved pit latrine Pit latrine with slab	7 (70.0) 3 (30.0) 0 (0.0) 0 (0.0)	1 (100.0) 0 (0.0) 0 (0.0) 0 (0.0)	0 (0.0) 0 (0.0) 1 (25.0) 1 (25.0)	0 (0.0) 1 (50.0) 0 (0.0) 1 (50.0)
Toilet facility is shared with other facilities	0 (0.0)	0 (0.0)	2 (50.0)	0 (0.0)

*CRHC* Community Rural Health Centre, *MH* Mission Hospital

Maternity users, SMAG/Neighbourhood Health Committee members and elderly women participants believed that the lack of electricity for lighting as well as water and sanitation facilities also prevented use of maternity homes. While all maternity homes, with the exception of 27% of CRHC homes, were connected to the national electrical grid, 40% of CRHC homes did not have electricity on the day of the assessment. CRHC in-charges explained that electricity interruptions were common due to load shedding and delayed payment of electricity bills by district offices. Only the two mission hospitals had backup generators. Access to water and sanitation was also limited. With respect to sanitation, only one mission hospital maternity home and 53% of CRHC homes had flush toilets. Maternity homes at mission hospitals and only 21% at CRHCs, had piped water. Limited water access led to quick departures following delivery:

*When I delivered last year, I went home immediately […] it was impossible to keep myself clean without water in the maternity ward and maternity home despite the midwife advising me to stay until the following day (Woman who gave birth at a CRHC).*

Maternity home users, SMAG/Neighbourhood Health Committee members and elderly women participants also identified amenities they thought were important for maternity homes to provide, namely security and privacy, food, and transportation. A CRHC maternity home user said she preferred giving birth at one of the mission hospitals where there was a fence, for security reasons. She suggested having a watchman at night for protection. Only homes at mission hospitals were fenced. She explained:

*Oftentimes, drunken men from the bars across the road come near the maternity home at night and this puts women at risk of rape (CRHC maternity home user).*

The intergenerational mixing of women in maternity homes was of concern because of its implications for privacy:

*There are different ages there who are waiting [for delivery]; so this older woman here undresses, a young lady is there seeing the nakedness of the mother. Such things will also deter people [from using maternity homes]. Placing curtains and secure windows and doors would improve privacy (District Community Nursing Officer).*

All maternity homes at mission hospitals and 50% of those at CRHCs provided food to pregnant women, but not to their companions.^1^ A district manager explained that the government grant excluded funds for maternity homes. Thus, many women at maternity homes had to cook for themselves, in separate kitchens or outdoors. Husbands, family members or SMAGs provided some food for pregnant women. One pregnant woman summarised what would attract her to use the maternity home at her facility:

*The thing that can make me go and wait at the maternity home is that it should be beautiful, good food should be available for pregnant women, and there should be enough beds. Comfort can make us go and wait at the maternity home. The maternity home should be well plastered and entertaining (Woman who gave birth at a CRHC).*

Bicycles were the most commonly used form of transport for pregnant women due to the high cost of motorized transport and the poor condition of roads. One community hired a vehicle or a bicycle to transport pregnant women. In other communities, husbands or families hired vehicles or bicycles individually. However, the trip was sometimes difficult:

*My husband hired a bicycle from a neighbour to come and collect me from the hospital but I could not manage to ride on it with a baby. We put my luggage at the back of the bicycle and walked home. Our village is about 2 h walk to this hospital (Woman who gave birth at a mission hospital).*

### Use of maternity homes

On the day of data collection in Luapula Province, an average of 12 pregnant women and eight companions were staying at maternity homes at mission hospitals and 0.9 pregnant women and 0.9 companions at maternity homes at CRHCs. Only five of 17 facilities had maternity home registers and could report use levels over the past 3 months as registries had recently been started. Use had increased over the preceding 3 months. On average, 24 pregnant and postpartum women and 25 companions had stayed in mission hospital maternity homes in the past 3 months.

Most maternity home users, non-maternity home users, SMAGs members and senior women thought the homes benefitted women who: lived far from the health facility, had pregnancy complications or HIV, were older and had many previous births, were pregnant for the first time or adolescents, or had a history of caesarean sections or complications during pregnancy or delivery. They agreed that maternity homes are the best place where pregnant women could wait for delivery because facilities offer: (a) skilled maternal care from nurses, midwives, or clinical officers; (b) adequate management of complications; (c) opportunity for a referral when health centre staff cannot manage a maternal complication; and (d) postpartum uterotonics to help contract the uterus and stop bleeding.

Traditional leaders and SMAGs also promoted use of maternity homes and facility-based births. In fact, some traditional leaders charged penalties in the form of cash or livestock^2^ to women who gave birth at home. These penalties were agreed upon at community meetings that included community members, the chief or village headman, as well as healthcare workers from the facilities in some instances. While these penalties were not consistently enforced, they effectively deterred some women from delivering at home. Nonetheless, some women still gave birth at home for fear of being shamed or criticised by health workers if they had no husbands or husbands who failed to provide supplies, such as baby layettes or bleach, which the facility was often lacking. A mother who gave birth at home explained:

*Some health workers shout at women for not bringing a baby layette or jik [bleach] to the maternity ward. Some husbands cannot afford these items due to high poverty in this province (non-maternity home user to Non-maternity home user).*

Additional reasons for giving birth at home included distance, transport costs to the facility, past favourable experiences with home birth, lack of a separate maternity ward and little privacy at the facility, shortage of trained staff, discomfort with male providers, unprofessional care from health providers including disrespectful attitudes towards women or failures to keep health information confidential. A male spouse acknowledged that the health facility was the best place to deliver but described some of the health staff as arrogant and uncaring.

Despite these concerns, many women in Luapula Province reported that they would use maternity homes in the future. The study’s field guide had a question about willingness to pay for staying at the maternity home, and some women indicated a willingness to contribute one to five Kwacha (2013 exchange rate: $0.20–$1.20) for their stay at the maternity home. A traditional leader believed that with sensitisation, some people could contribute up to one Kwacha per month toward the upkeep of the maternity home. However, most participants were sceptical that women would pay for maternity homes because the health system services for RMNCH are free of charge, by policy, in Zambia. A partner agency respondent explained, “if mothers are asked to pay [for maternity care], very few [would] come [to maternity homes].”

### Relationships among maternity homes, facilities and communities

Community groups played an active role in the development, construction and operation of maternity homes. SMAG/Neighbourhood Health Committee members with support from chiefs and faith-based organizations had mobilized to build the homes and provide transport for pregnant women, including during emergencies after delivery. SMAGs often played that role, and Neighbourhood Health Committees and traditional birth attendants mobilized communities in health zones where no SMAGs existed. At most homes, community groups also supported women during pregnancy, labour and delivery and ensured effective communication between the community and health staff. A SMAG member explained:

*We do not allow pregnant women to deliver at home. We advise those who are about to deliver to go to the maternity home to wait for delivery. In our zone, we have a timetable for SMAGs to accompany pregnant women to the maternity home. Sometimes, two people accompany the women but only female members stay with the woman at the maternity home until she delivers and take her back home (SMAG member).*

Community members also participated in the homes’ maintenance. While government employees were responsible for cleaning maternity homes at mission hospitals and 60% of CRHCs, companions (15%), SMAGs (9%), pregnant women (9%) and volunteers (7%) also cleaned the homes.

Health workers facilitated the work of the community groups. Health workers in Luapula Province trained the SMAGs in RMNCH, HIV and community mobilisation using the National Safe Motherhood Action Group Training Manual [30]. Then the SMAGs conveyed messages about antenatal care, danger signs in pregnancy, and prevention of mother-to-child transmission of HIV to community members and encouraged use of maternity homes and facility-based births.

The SMAG/Neighbourhood Committee members, elderly women, chiefs and village headmen recommended communities could support maternity homes in additional ways. Maternity home users, SMAGs and elderly women suggested they organise activities to occupy women’s time, while women and most community groups proposed teaching pregnant women income-generating skills, such as sewing, gardening and fish farming.

While community members assisted health workers with weighing, immunizing children, and organizing educational talks in antenatal, family planning and postnatal care clinics, District Medical and Nursing Officers reported that they themselves did not perceive management of maternity homes to be part of their responsibilities. Hence, SMAG/Neighbourhood Committee members managed the homes at CRHCs to fill that management gap. Furthermore, one maternity home in a mission hospital and only 36% of those in CRHCs had a formal administrative review system to monitor and evaluate maternity homes. None of them had maternity home operating protocols. Staff meetings to discuss the management of maternity homes were irregular.

### Successful practices in existing maternity homes models in Central Province

Well-functioning maternity homes in Central and Lusaka Provinces offer a positive model for other provinces. One proven practice in Central Province is involving community organizations in developing and operating maternity homes. In Serenje and Mkushi Districts, SMAGs raised and managed funds to sustain maternity homes, provided food for pregnant women, and taught them farming skills. Facility in-charges monitored their activities, including revenues and expenditures, and accountants from the district offices conducted audits to ensure accountability. The SMAGs also organized in-kind contributions: community members carried water and sand and moulded bricks to build maternity homes and kitchens.

Another successful practice is for health workers themselves to promote the use of maternity homes at their CRHC and build community support. Health workers and headmen met local chiefs to discus RMNCH problems specific to each chiefdom. Similar to Luapula Province, in Central and Lusaka provinces, chiefs then called stakeholder and community meetings to resolve the problems. Stakeholders agreed that all pregnant women should wait in maternity homes for childbirth in health facilities and home deliveries were fined in the form of cash or livestock. Payments made to chiefs were used for maternity home projects run by SMAGs. According to the 26 SMAG members who participated in the study, the community accepted the fines because they had participated in the decision to charge for home births. In addition, the SMAGs assisted vulnerable women to support their use of maternity homes by providing baby layettes. As a result, women increasingly used maternity homes even though they were overcrowded, poorly ventilated and lacked supplies.

Several shortcomings were also noted in Central and Lusaka Provinces. While the SMAGs were instrumental in the success of some maternity homes, their initiatives had finite timelines and funding. At the time of data collection, some of their projects had stalled. In addition, not all health zones had SMAGs to spearhead efforts promoting maternity homes. Also, the number of rooms at the four maternity homes in Serenje and Mkushi Districts did not meet the needs of these communities. For instance, most pregnant women from Mkushi used a maternity home in Rufunsa to avoid crossing the river without a bridge during the rainy season. As a result, the maternity home was always full. Transport to the health facility from rural homes therefore affected maternity home and health facility use.

## Discussion

In Luapula province, women used maternity homes despite deficiencies. This study provides one of the few in-depth looks at sustainable maternity home use in low-resource settings. It found that while women recognized the benefits of maternity homes, many CRHC maternity homes did not meet the needs and expectations of the community - addressing the first research question. They lacked sufficient rooms, supplies, electricity, water, and sanitation facilities; did not guarantee women’s safety; and did not provide food or transportation. Previous studies have also reported that these issues affect usage of maternity homes [9–14]. This study highlights differences between maternity homes at mission hospitals and CRHCs. The findings suggest that maintenance interventions, such as improved infrastructure, food, privacy and security could realize the full potential of maternity homes, especially for CRHC maternity homes. Simply budgeting for and constructing a maternity home is one step in reducing maternal and neonatal mortality but it does not ensure its use. Further research should investigate which amenities and elements of infrastructure are associated with higher maternity home use.

The financial cost of using maternity homes was raised as one of the main barriers to utilization - addressing research question 2. Other studies have found that cost is a barrier in Eritrea [9], Zimbabwe [12], and Kenya [13]. Women in the Eastern Province of Zambia who could not afford to purchase clothes and other items for their baby did not use maternity homes [17]. Our findings confirm that costs associated with transportation, meals, and goods, such as bleach and baby layettes, deter use. Notably, the communities surveyed in Luapula Province imposed financial or livestock penalties for home deliveries to motivate women to use maternity homes. This raises concerns regarding women’s rights to choose where and when to seek health care. The fines may also distract from more constructive strategies to encourage maternity home usage, such as addressing women’s limited decision-making power in the home and improving the quality of health and maternity home services.

At the time of the study, some maternity home users, SMAG members, chiefs and village headmen suggested that women might be willing to pay a fee to use maternity homes despite Zambia’s free healthcare system. This is no longer necessary since maternity homes have been integrated in the national health system and RMNCH services are free in Zambia. However, use levels will depend on availability of necessities and amenities acceptable to women and the community. The literature on maternity home fees is scant, and further investigation is needed to understand the appropriate size of fees and their effect on use.

Finally, the diversity of stakeholders who participated in this study allowed us to explore the relationship among communities and women, maternity homes and facilities - addressing research question 3. Community groups frequently assisted with the construction of maternity homes, provided food and transport, and advocated for use of the homes. Partnerships between communities and facilities often flourished after enlisting the support of chiefs or village headmen and relied on the activities of individual health workers, SMAGs and Neighbourhood Health Committees. A study conducted in rural Zambia in 2016 confirms these findings on the importance of integrating the community in planning and managing to ensure the acceptability and sustainability of maternity homes [8]. In a large study conducted in Ethiopia, the success of maternity homes was linked to their acceptance by and ties to the community [15]. Another study in Eritrea found encouragement and referral of women to maternity homes were influential factors in usage [9].

Existing partnership between health workers in rural facilities, the broader health system, women and their families and other community stakeholders in Zambia requires continuous commitment and engagement to ensure that maternity homes lead to reduced maternal and neonatal mortality and improved health outcomes. Examination of maternity homes from Central and Lusaka Provinces suggest caution about relying too heavily on the community. While their experience confirms that community engagement is critical to the development of maternity homes and their sustained success, community resources alone are inadequate and partnerships with community groups may not be sustainable. Ultimately, the sustainability of maternity homes requires the investment of government funding in addition to other sustainable funding sources including novel funding mechanisms to strengthen partnerships and ensure success on longer time scales and away from short project-based timelines.

### Limitations

The results of this study should be treated cautiously as they include the completed maternity homes in Luapula Province. The study assessed four of the province’s nine districts and 21 of its 68 facilities. These were, however, the only districts with maternity homes at the time of the study. Similarly, the success stories from Central and Lusaka Provinces draw on only three facilities located in districts that may differ somewhat from those in Luapula. The inclusion of ten husbands in the sample provides a male perspective missing in most studies of maternity homes, but conducting husbands-only focus group discussions or increasing the number of men in a similar study could offer a more balanced view of maternity homes and their place in the community. Further studies are needed to understand the role of male participation in promoting, supporting, and sustaining maternity homes. In addition, data from healthcare workers who provide direct care to the women in maternity homes was not collected and would likely provide a distinct perspective on barriers and facilitators that affect use of homes.

## Conclusions

The findings from four districts in Luapula Province juxtaposed with three success stories outside the province provide key insights for the continued promotion of maternity homes in Zambia. Attention needs to be paid not only to the construction of maternity homes at rural health facilities, but also to their continuous maintenance and adherence to standards in their maintenance and operations. The success and sustainability of maternity homes require strong commitment and support from all stakeholders, especially in the community but also including the Zambian government. Nevertheless, sustaining existing community commitment to maternity homes will require more than government funding. As this evaluation has shown, policy and program evaluations are essential to strengthen, define, and streamline maternity homes’ functions, partnerships, and success.

## Additional files

Additional file 1: Field Guide: Focus Group Discussions and Key Informant Interviews – documents used by interviewers to guide the different focus group discussions and key informant interviews. (DOCX 64 kb)

Additional file 2: Maternity Home Assessment Tool – document used to collect data about the structure and amenities available at each maternity home. (DOC 219 kb)

Additional file 3: Service Abstraction Form – document used to extract data on deliveries from maternity home registers. (DOC 31 kb)

## Abbreviations

CRHC: Community Rural Health Centre
RMNCH: Reproductive, Maternal, Neonatal and Child Health
SMAG: Safe Motherhood Action Group

## Footnotes

1 **On companions**: Companions are typically female relatives or neighbours or members of the SMAGs who accompany pregnant women to maternity homes, stay with them, assist them as needed and take them home after the facility-based birth. In some cases, spouses accompany the women to maternity homes.

2 **On penalties**: All the communities surveyed in Luapula and Central Provinces required women to stay in maternity waiting homes prior to delivery and enacted fines agreed between the chiefs and communities on women or families who did not comply.
