# Supplementary material for: Experiences with and expectations of maternity waiting homes in Luapula Province, Zambia: a mixed–methods, cross-sectional study with women, community groups and stakeholders
Source: BMC Pregnancy Childbirth. 2018 Jan 25;18:42. doi: 10.1186/s12884-017-1649-1 (PMC5785796; doi:10.1186/s12884-017-1649-1)
Supplement: Supplementary file 1 — Field Guide: Focus Group Discussions and Key Informant Interviews – documents used by interviewers to guide the different focus group discussions and key informant interviews. (DOCX 64 kb) [file 12884_2017_1649_MOESM1_ESM.docx]

**FIELD GUIDE – FOCUS GROUP DISCUSSIONS**

| **Group 1:** Women (including pregnant women) who delivered last time in a health facility and USED a maternity waiting home (“mothers’ shelter”)  **Group 2:** Women (including pregnant women) who delivered last time in facility, and did not use a maternity waiting home (“mothers’ shelter”)  **Group 3:** Women who delivered at home or TBA in last birth OR pregnant women who will be first-time mothers |
| --- |

**Introduction:** Thank you for speaking with us today. We are trying to improve or create mothers’ shelters in this community. A mothers’ shelter is also called a maternity waiting home. It is a place near a health facility for pregnant women to stay and wait until it is time to give birth. Let’s go around the room and introduce ourselves.

| **Group** | **Q. No.** | **Question** |
| --- | --- | --- |
| 1,2,3 | 1 | **Briefly**, please tell me about the customs and traditions in your community for pregnant women, women giving birth, and the few days after delivery.  PROBE FOR:   1. Who is usually present during labor? What does this person do for the mother while she is in labor? 2. Who is usually present at the delivery? What does this person do for the mother during the delivery? 3. Is there a different person to tend to the newborn other than the person tending to the mother? Who is that person (s)? 4. What are the common things you do for the mother and the baby immediately after birth? In the following week? 5. How long should a woman stay at (identified facility) after having a baby? |
| 1,2,3 | 2 | Please tell me about the circumstances of your most recent pregnancy and delivery.   - 1. What was the pregnancy like?   2. What were your plans about where to deliver the baby (in a facility or at home)?   3. What were the important factors you thought about in determining where to give birth?   4. Who helped you make this decision and/or gave you permission. |
| 1 | 3 | Describe how you decided to stay at the mothers’ shelter before delivering. How did you learn about it?  PROBE FOR:   - 1. Most important reasons for making this decision,   2. People who helped make the decision and/or gave permission.   3. Reasons she felt reluctant to stay at the mothers’ shelter. |
| 1,2,3 | 4 | What **arrangements did you make at home** to have someone take over your responsibilities during and immediately after the birth? |
| 1,2 | 5 | Tell me about how you **traveled** to the mothers’ shelter or health facility.  PROBE FOR:   1. Distance 2. Length of time 3. Means of transport 4. People that accompanied |
| 1 | 6 | **How early** (number of days or hours) before the baby was born did you arrive at the mothers’ shelter? What things (factors) affected your coming to the mothers’ shelter at the time that you did? |
| 1 | 7 | Some mothers’ shelters provide **amenities** but sometimes pregnant women have to bring their own **supplies**. I haven’t seen the mothers’ shelter where you stayed. Could you tell me what it is like?   - 1. What amenities and supplies were there? How satisfied were you with them?   2. What did you have to bring or buy in order to stay there?   3. Probe specifically for issues related to food and cooking, if not already discussed.   4. Probe for whether another person (friend, relative) stayed with them at the mothers’ shelter |
| 1 | 8 | What kind of employees were **on staff** at the mothers’ shelter? How satisfied were you with their performances and their manner? |
| 1 | 9 | How did you occupy your time while waiting at the mothers’ shelter?   - 1. How did you feel during this time?   2. What was it like staying with the other pregnant women? |
| 1 | 10 | How would you feel about being taught a skill while staying at the mothers’ shelter?   1. What types of skills would be useful? 2. How you feel about learning new farming and gardening methods? 3. Are there other types of information which would be useful? |
| 1 | 11 | We would like to get your advice on how to improve the mothers’ shelters. What would you recommend in terms of the facility, the amenities and supplies, or anything else? |
| 1 | 12 | How, in your opinion, can the **community, including mothers and families, support** the mothers’ shelters? |
| 1 | 13 | What, if anything did you or your family **pay for** at the mothers’ shelter, for example, fees or paying for supplies?  PROBE FOR:   1. Paid with cash or with in-kind donations? 2. Who collected this payment? |
| 1 | 14 | For those who delivered at the facility, to what extent did the mothers’ shelter seem **linked or related to the health facility? For example, were** staff members the same at both places? |
| 1,2 | 15 | Please tell me about the **birth** of your baby.   - 1. How satisfied were you with the setting? (comfort, cleanliness, etc.)   2. Who was with you? (Family, health care providers, etc.)   3. How satisfied were you with the provider or providers?   4. How did this compare to your previous birth experiences, either your own or experiences you have heard from other women? |
| 1,2 | 16 | How long did you **stay at the facility** after the baby was born? Was the baby with you at all times or somewhere else? |
| 1 | 17 | What was the **cost of delivery care**? |
| 1 | 18 | Did you **return** to the mothers’ shelter after the birth for some time?   1. How long? 2. Did you receive postnatal care? |
| 1 | 19 | How did you **return home** from the mothers’ shelter and facility? |
| 1 | 20 | **Closing:** Those are all the questions that I had planned to ask. Are there other things that are important for me to know about the mothers’ shelter or the facility attached to it [or, mention facility by name]? |
| 3 | 21 | In your opinion, what are the **disadvantages** of giving birth in the facility for women in your community?  PROBE FOR:   - 1. Comfort/cleanliness/structure   2. Amenities and supplies   3. Cost |
| 3 | 22 | In your opinion, what are the **advantages** of giving birth in the facility for women in your community? |
| 2,3 | 23 | What have you been told about the mothers’ shelter?  PROBE FOR:   - 1. Amenities, facility, etc.   2. Payments and fees associated with using it   3. Women’s personal experiences while using it |
| 2,3 | 24 | Who (if anyone) in your community **encouraged** you to use a mothers’ shelter?   - 1. What did **community health workers** say, if anything, regarding the mothers’ shelter?   2. What did family members say? |
| 2,3 | 25 | In your opinion, what type of women would benefit from using the mothers’ shelter?  PROBE FOR:   1. Need based on health reasons 2. Need based on geography 3. Need based on socioeconomic factors |
| 2,3 | 26 | How would you feel about staying at the mothers’ shelter during the last days of pregnancy? |
| 2,3 | 27 | What can the **community including mothers and families, do to support** the mothers’ shelters? |
| 2,3 | 28 | Is there anything else you would like to say about this subject? |
| 2,3 | 29 | I mentioned in the beginning that a mothers’ shelter is a place for pregnant women to stay while awaiting the time to give birth. How would you feel about staying at the mothers’ shelter during the last days of pregnancy?  PROBE FOR:   1. Feasibility 2. Arrangements that would need to be made 3. Support/permission to be obtained |
| 2,3 | 30 | What would the essential elements be that would make a mothers’ shelter acceptable for pregnant women and their families?  PROBE FOR:   - 1. Aspects of the structure   2. Amenities, furnishings, supplies   3. Issues related to food/cooking   4. Kind of staff on hand to assistant women   5. Other factors |
| 2,3 | 31 | What kinds of family support would pregnant women need to obtain before using the mothers’ shelter?  PROBE FOR:   - 1. Whose support/permission would be needed   2. Help with childcare/housekeeping during the absence   3. Help with transport   4. Help with costs   5. Person to accompany |
| 2,3 | 32 | How would you feel about being taught a skill while staying at the mothers’ shelter?   1. What types of skills would be useful? 2. How you feel about learning new farming and gardening methods? 3. Are there other types of information which would be useful? |
| 2,3 | 33 | **Let us imagine that the mothers’ shelter has already been built.** How would people get information about the women’s shelter? Who would be the best people in your community to encourage women to use the mothers’ shelter? |
| 2,3 | 34 | What could the **community do to support** the mothers’ shelters? |
| 2,3 | 35 | How much would people be willing to pay per day to use the mothers’ shelters? |
| 2,3 | 36 | Is there anything else you would like to say about this subject? |

| **Group 4:** Elderly women |
| --- |

**Introduction:** Thank you for speaking with us today. We are trying to improve or create mothers’ shelters in this community. A mothers’ shelter is also called a maternity waiting home. It is a place near a health facility for pregnant women to stay and wait until it is time to give birth. Let’s go around the room and introduce ourselves.

| **Q. No.** | **Question** |
| --- | --- |
| 1 | Tell me about the **customs and traditions** in your community for pregnant women, women giving birth, and the few days after delivery.   - Who is usually present during labor? What does this person do for the mother while she is in labor? - Who is usually present at the delivery? What does this person do for the mother during the delivery? - Is there a different person to tend to the newborn other than the person tending to the mother? Who is that person (s)? - What are the common things you do for the mother and the baby immediately after birth? In the following week? - How long should a woman stay at (identified facility) after having a baby? |
| 2 | **Where** do most pregnant women in your community deliver their babies? When your daughter/daughter-in-law has been pregnant, what kinds of discussions have you had with your family about **where** she should deliver and who should attend the birth? |
| 3 | In your opinion, why do some pregnant women **deliver at home?**  Probe for issues related to:   - Access (distance, transport, cost) - Permission or support from family members - Household responsibilities - Perceptions of health facility and quality of care - Perception of pregnant woman’s health risk - Myths or misconceptions about home or facility delivery |
| 4 | In your opinion, why do some pregnant women **deliver at a facility**?  Probe for issues related to:   - Access (distance, transport, cost) - Permission or support from family members - Household responsibilities - Perceptions of health facility and quality of care - Perception of pregnant woman’s health risk - Myths or misconceptions about home or facility delivery |
| 5 | In your opinion, where is the **best place** for pregnant women in your community to give birth? Why?   - What are the advantages of delivering at this place (identified location)? - What are the disadvantages of delivering at this place (identified location)? |
| 6 | In your opinion, **how** could pregnant women in your community **benefit** from using a mothers’ shelter? Who would use the shelter most? Please explain.  PROBE FOR:   - Need based on health reasons - Need based on geography - Need based on socioeconomic factors - Need based on gravida - Previous experience with birth in the facility |
| 7 | In your opinion, what are the **reasons pregnant women might use or not use a mothers’ shelter**? (PROBE FOR: social, cultural, economic structural factors.  PROBE FOR:   - Social/cultural reasons - Financial implications - Perceptions of health and personal safety - Permission/support from family - Encouragement or discouragement from peers |
| 8 | What kind of **support from families or to families** would pregnant women need to obtain before using the mothers’ shelter?  PROBE FOR:   - Whose support/permission would be needed - Help with childcare/housekeeping during the absence - Help with transport - Help with costs - Person to accompany |
| 9 | What do you imagine **it would be like** if your daughter or daughter-in-law went away for the last weeks before having a baby?  PROBE FOR:   - How would it affect you? - How would it affect the household of your daughter or daughter-in-law? |
| 10 | Which persons should be allowed to **stay with** pregnant women in the mothers’ shelter?  PROBE FOR:   - Children, including number and age - Other family members - Traditional birth attendants - Other support persons |
| 11 | If you were considering letting your daughter or daughter-in-law stay at the mothers’ shelter, what **amenities would be required**?  PROBE FOR:   - Aspects of the structure - Amenities, furnishings, supplies - Issues related to food/cooking - Types of staff on hand to assistant women - Other factors |
| 12 | I also want to get your opinion on an idea that we have. We are considering teaching women some **skills** or providing other information to women-- while they are staying in the mothers’ shelter.   - What kind of skills would be useful, in your opinion? - How would you feel about your daughter or daughter-in-law learning new farming or gardening methods? - What kinds of information would be useful, in your opinion? - Do you have other ideas about how to help women pass the time during their stay at the mothers’ shelter? |
| 13 | Why might a pregnant woman in your community who is high-risk or who lives far from the facility **choose not to stay** in the mothers’ shelter before delivery?   - What things make it hard for a woman to use the mothers’ shelter? - What things make it easy for a woman to use the mothers’ shelter? |
| 14 | What could your **community do to support** the mothers’ shelters? |
| 15 | Who is responsible for the **day-to-day management** of the mothers’ shelter in your community? |
| 16 | What are the types of **activities** this (person/group) does?  How is this working? |
| 17 | How do women, families, and community members **contribute to maintenance**/ upkeep of the mothers’ shelter? Can you describe some of the activities? |
| 18 | Who provides the **resources** (e.g. food, money, etc.) to keep the mothers’ shelter open? What resources does the community contribute to the running or operation of the mothers’ shelter in your area? |
| 19 | What has been the greatest **challenge** associated with keeping a mothers’ shelter open? What can be done to **overcome** these challenges? |
| 20 | Do you have any other thoughts to share about this subject? |

| **Group 5:** Community group members |
| --- |

**Introduction:** Thank you for speaking with us today. We are trying to improve or create mothers’ shelters in this community. A mothers’ shelter is also called a maternity waiting home. It is a place near a health facility for pregnant women to stay and wait until it is time to give birth. Let’s go around the room and introduce ourselves.

| **Q. No.** | **Question** |
| --- | --- |
|  | Briefly, please tell me about the **customs and traditions** in your community for pregnant women, women giving birth, and the few days after delivery. |
|  | Please describe how women and families in your community decide **where** a pregnant woman will deliver her baby. |
|  | In your opinion, why might a woman decide **not** to deliver at the health facility? |
|  | What would make it **easier or more desirable** for pregnant women to deliver at the health facility? |
|  | In your opinion, where is the **best place** for pregnant women in your community to give birth? Why?   1. What are the advantages of delivering at this place (identified location)? 2. What are the disadvantages of delivering at this place (identified location)? |
|  | If there were a mothers’ shelter at the health facility (where pregnant women who have a high risk of health problems or live very far away could stay in the weeks before their delivery), how would this **affect women’s decision** whether or not to deliver at the facility? |
|  | If there were/is a mothers’ shelter at your local health center, why might a woman who is high-risk or who lives very far from the facility **choose not to stay** in the shelter before delivery? |
|  | In your opinion, what **features** should be included in a mothers’ shelter in order to encourage women to use it?   - - *Probe:* If the community has a mothers’ shelter, ask which features it currently has and how it compares to what is desirable |
|  | How does/can your **community support** the mothers’ shelter, so that it does not depend on funds from the government or other donors?   - - *Probe* about how the community can ensure inclusion of the features noted in Q6 |
|  | If the mothers’ shelter had [*note features listed in Q6*], do you think women would be **willing to pay** to stay at the shelter?   - - *Probe:* Why or why not? If yes, what cost do you think is reasonable? |
|  | In your opinion, what **challenges** do you see/foresee in maintaining a mothers’ shelter with the features you’ve described? |
|  | Please describe how you think the mothers’ shelter could best be **managed** and by whom.   - - *Probe:* What role each NHCs, SMAGs, CHAs and other CHVs could play; if and what types of compensation the groups or other individuals would require |
|  | Does your community or community groups have the capacity to **manage gardens** which could be used to feed women at the mothers’ shelter?   - - *Probe:* Who in the community/groups has technical agricultural knowledge; how gardens could be managed? |
|  | Do you have any other comments about pregnancy and delivery or mothers’ shelter in your community? [Pause] Thank you for your time. |

**FIELD GUIDE – KEY INFORMANT INTERVIEWS**

| **Group 1:** Traditional leaders (chiefs and village headmen) |
| --- |

**Introduction:** Thank you for speaking with us today. We are trying to improve or create mothers’ shelters in this community. A mothers’ shelter is also called a maternity waiting home. It is a place near a health facility for pregnant women to stay and wait until it is time to give birth.

1. Your Royal Highness, for how long have you been Chief/Headman in your kingdom?
2. Your Royal Highness (to the Chief)/Village headman/woman), please describe your role in ensuring the health of the members of your kingdom, particularly women around the time of childbirth and newborns.
   - What has been your role in health campaigns? (e.g. to reduce maternal mortality and VMMC)
3. Please describe any strategies that your community uses to encourage or help women to go to the facility for delivery.
   - As Chief/Headman, has your office encouraged women to deliver at facilities? In what way?
   - Have any financial or in-kind resources help cover the costs of supporting women to deliver in facilities, or stay at mothers’ shelters?
4. In your opinion, how could pregnant women in your community benefit from using a mother’s shelter? Who would use the shelter most? Please explain.

**PROBE FOR**:

- - Need based on health reasons
  - Need based on health reasons
  - Need based on geography
  - Need based on socioeconomic factors
  - Need based on gravida
  - Previous experience with birth in the facility

1. What kind of **support from husbands/families or to husbands/families** would pregnant women need to obtain before using the mother’s shelter?

**PROBE FOR**:

- - Whose support/permission would be needed
  - Help with childcare/housekeeping during the absence
  - Help with transport
  - Help with costs
  - Person to accompany
  - Any other issue related to male and female (gender) roles within households or communities.

1. What would you do Your Royal Highness/Headman, to improve the support in your community for mother’s shelters? (Community: “What can the community do to support the mother’s shelters?”
   - Management: “How could the mother’s shelters be better managed?
   - Coordination: “How could health facilities better work with mother’s shelters?”
2. What do you think is the appropriate role for the community in supporting the mothers’ shelters?
   - How can community support be generated?
   - Are there other strategies that you could imagine that would bring more resources to the mother’s shelter?
3. Do you think families would be willing to pay (money or in-kind donations) to use the mother’s shelter? If so, how much?
4. Is there anything else you would like to tell me about mother’s shelters or maternity waiting homes in your community?

| **Group 2:** Health facility in-charges |
| --- |

**Introduction:** Thank you for speaking with us today. We are trying to improve or create mothers’ shelters in this community. A mothers’ shelter is also called a maternity waiting home. It is a place near a health facility for pregnant women to stay and wait until it is time to give birth.

1. What’s a typical day like for maternity care services at your facility?
2. What do you see as the strengths and weaknesses of maternity care services at your facility?
3. We know that not all women in this district seek routine delivery care at health facilities.

- In general, what do you think are the main factors that prevent women from delivering at this health facility?
- What are the factors that lead them to give birth at home or with a traditional birth attendant or relative?

1. We will discuss mother’s shelters later. Other than mother’s shelters, what kinds of policies or programs do you think would be most helpful to encourage women to deliver at a health facility?
2. What kinds of local traditions related to pregnancy and childbirth are accommodated within the health facility?
3. Why do women decide to deliver at this health facility?

Probe for issues related to:

- Access (distance, transport, cost)
- Permission or support from family members
- Household responsibilities
- Perceptions of health facility and quality of care
- Perception of pregnant woman’s health risk
- Myths or misconceptions about home or facility delivery

1. In your opinion, how could pregnant women in your community benefit from using a mother’s shelter? Who would use the shelter most? Please explain.

Probe for:

- Need based on health reasons
- Need based on geography
- Need based on socioeconomic factors
- Need based on gravida
- Previous experience with birth in the facility

1. How could providers at the health facilities benefit from having women stay at the mother’s shelter?
2. What kind of **support from families or to families** would pregnant women need to obtain before using the mother’s shelter?

Probe for:

- Whose support/permission would be needed
- Help with childcare/housekeeping during the absence
- Help with transport
- Help with costs
- Person to accompany

1. Which persons should be allowed to stay with pregnant women in the mother’s shelter?

Probe for:

- Children, including number and age
- Other family members
- Traditional birth attendants
- Other support persons

1. What would be the essential things (elements) that would make a mother’s shelter good for pregnant women and their families?

Probe for:

- Aspects of the structure
- Amenities, furnishings, supplies
- Issues related to food/cooking
- Types of staff on hand to assistant women
- Other factors

1. How could women, families, and community members contribute to maintenance/upkeep of mother’s shelter?

- Can you describe some of the activities?

1. What are the best ways that mother’s shelters could be linked to the health facility/labor and delivery ward?

Probe for:

- What policies and/or guidelines need to be in place?
- Transfer to facility: how does the transfer from mother’s shelter to facility happen prior to or during labor so women can receive timely care?
- Families – how can family or support members be accommodated in mother’s shelters AND during labor and delivery?

1. How could costs of the mother’s shelters and costs of the facility be supported and sustained?

**[QUESTIONS 15-21 FOR COMMUNITIES WITH MOTHER’S SHELTERS]**

1. Who is responsible for the day-to-day management of the mother’s shelter in your community?
2. What are the types of activities this (person/group) does?

- Can you tell me how you think this is working?

1. Who provides the resources (e.g. food, money, etc.) to keep the mother’s shelter open?

- What resources does the community contribute to the running or operation of the mother’s shelter in your area?

1. What has been the greatest challenge associated with keeping a mother’s shelter open?

- What can be done to overcome these challenges?

1. How do mothers’ shelters help meet health facility goals? Does the population use the mothers’ shelters? Why or why not?
2. Are there shared personnel or volunteers between the mothers’ shelter and the health facility?
3. Where do women and their families get food and water while staying at the mother’s shelter?

- How about at the health facility?
- What about the providers – where do they get water and food while working at the facility?

| **Group 3:** District Community Medical Officers / District Community Nursing Officers |
| --- |

**Introduction:** Thank you for speaking with us today. We are trying to improve or create mothers’ shelters in this community. A mothers’ shelter is also called a maternity waiting home. It is a place near a health facility for pregnant women to stay and wait until it is time to give birth.

1. How many **health facilities of each type/level** are in this district?

- How many health facilities in your district have maternity wards?
- How many of these facilities are operational?
- How many beds are in each of these facilities?

1. How many **health providers of each cadre** work in maternity in the district?

- In general, how many years have these providers been in these posts?
- Are the providers from this district?

1. How many **community based health volunteers (CHV) by group** promote maternal and newborn health in maternity in the district?

- In general, how many years have these community based volunteers had this role?
- What are their responsibilities?
- How many health facilities work with or are supported by neighborhood health committees (NHCs)?
- How many communities have functioning safe motherhood action groups (SMAGs)?

1. What is the **skilled birth attendance rate**? (delivery with skilled attendant in facility or otherwise) Does this vary *within* the district?

- What is the ***maternal mortality ratio*** for the district?
- Newborn mortality rate?

1. What **goals** does your district need to reach regarding deliveries and maternal and newborn health? (per National Health Strategic Plan 2011 - 2015)
2. Which facilities **currently have** mother’s shelters? What purpose do the mother’s shelters serve? How does the existence of the mother’s shelters affect the skilled birth attendance rate?
3. How do mothers’ shelters help **meet district health goals**? Does the population use the mothers’ shelters? Why or why not?

Probe for:

- In your opinion, what are the **challenges and disadvantages** of the mothers’ shelter?

1. How does the *facility in-charge* view the mother’s shelter and how it may help or hinder the work of the facility? How do the *health care providers* view the mother’s shelter? [*Omit question if we are asking these questions to these actual people*?]
2. Are there **shared personnel** or volunteers between the mothers’ shelter and the health facility?
3. Are there any **shared supplies or amenities** between the mothers’ shelter and the health facility? What about water and food for the providers? (*Water and food for the mothers and families*)
4. What are the **needs** of the facilities’ maternity wards and the needs of the mothers’ shelters? Are there any shared needs?
5. What are the **best ways** that mother’s shelters can be linked to the health facility/labor and delivery ward?

As needed, probe for:

- What policies and/or guidelines need to be in place?
- Staffing of the mother’s shelter, and of the health facility – do any overlap? (shared) How does staff/personnel/ and volunteers work together and communicate?
- Transfer to facility: how does the transfer from mother’s shelter to facility happen prior to or during labor so women can receive timely care?
- Supplies and amenities (& equipment?): how can these be assured at the mother’s shelter AND at the facility to meet clinical needs as well as personal needs? (food, clean water)
- Families – how can family or support members be accommodated in mother’s shelters AND during labor and delivery?
- **Sustainability and Costs:** how can costs of the mother’s shelters and costs of the facility be supported and sustained? Are there any joint mechanisms? What can the community do to support the mother’s shelter? And the health facility?

1. In your opinion, what would be the essential elements that would make a mother’s shelter acceptable for pregnant women and their families?
2. What would make them acceptable to the health providers in the nearby facility?

**Probe for:**

- Aspects of the structure
- Amenities, furnishings, supplies
- Issues related to food/cooking
- Types of staff on hand to assistant women
- Other factors

1. Is there anything else you would like to tell me about mother’s shelters or maternity waiting homes in your community?

| **Group 4:** Maternal health implementing partners |
| --- |

**Introduction:** Thank you for speaking with us today. We are trying to improve or create mothers’ shelters in this community. A mothers’ shelter is also called a maternity waiting home. It is a place near a health facility for pregnant women to stay and wait until it is time to give birth.

1. Ho Please describe the role of your organization as it relates to maternal and neonatal health. ***Probe*** for organization goals, objectives, activities in MNH
2. In your opinion, what are the biggest challenges in improving maternal health in Mansa and/or Samfya Districts? (*Core Q16*) ***Probe***: What do you think are the primary challenges to increasing the percentage of deliveries occurring in facilities?
3. What are the potential solutions/strategies to address these challenges?
4. What is your experience with mother’s shelters in this or other districts in Zambia?

**PROBE FOR**:

- Successes & challenges
- Impact of mother’s shelters on facility delivery
- Relationship between facility and mother’s shelter
- Relationship between mother’s shelter and community

1. Based on your experience, why might a woman who is high-risk or who lives far from the facility choose not to stay in the mother’s shelter before delivery? (*Core Q11)*
2. What do the health facility in-charges or providers do about this?
3. What have the communities been doing to encourage women to deliver in a health facility?
4. In your opinion, where is the ideal place for pregnant women in your community to give birth? Why? (*Core Q5)*

**PROBE FOR**:

- Advantages of a mother’s shelter
- Disadvantages of a mother’s shelter

1. In your opinion, what would be the essential elements that would make a mother’s shelter acceptable for pregnant women and their families? What would make them acceptable to the health providers in the nearby facility? (*Core Q10)*

**PROBE FOR**:

- Aspects of the structure
- Amenities, furnishings, supplies
- Issues related to food/cooking
- Types of staff on hand to assistant women
- Other factors

1. If the mother’s shelter had most or all of the ideal features you described, do you think women would be willing to pay to stay at the shelter?

**Probe**: Why or why not? If yes, what cost do you think is reasonable?

1. Sustainability and Costs: how can costs of the mother’s shelters and costs of the facility be supported and sustained? Are there any joint mechanisms?
2. What are the **best ways** that mother’s shelters can be linked to the health facility/labor and delivery ward?

**PROBE FOR**:

- What policies and/or guidelines need to be in place?
- How does facility staff oversee/support the shelter? How does staff/personnel/ and volunteers work together and communicate?

1. Transfer to facility: how does the transfer from mother’s shelter to facility happen prior to or during labor so women can receive timely care?
2. Supplies and amenities: how can these be assured at the mother’s shelter AND at the facility to meet clinical needs as well as personal needs? (Food, Clean water)
3. Families: How can family or support members be accommodated in mother’s shelters and during labor and delivery?
4. What do you think is an appropriate role for the facility in supporting the mothers’ shelters?
5. What do you think is a realistic role for the community in supporting the mothers’ shelters?
6. How can community support be generated?
7. Are there other strategies that you could imagine that would bring more resources to the mother’s shelter?
8. Is there anything else you’d like to tell me about mother’s shelters?

| **Group 5:** Couples |
| --- |

**Introduction:** Thank you for speaking with us today. We are trying to improve or create mothers’ shelters in this community. A mothers’ shelter is also called a maternity waiting home. It is a place near a health facility for pregnant women to stay and wait until it is time to give birth.

1. In your opinion, where is the **best place** for you/your wife to give birth? Why?

- What are the advantages of delivering at this place (identified location)?
- What are the disadvantages of delivering at this place (identified location)?

1. In your opinion, how could pregnant women in your community **benefit** from using a mother’s shelter. Who would use the shelter most? Please explain.

**PROBE FOR**:

- Need based on health reasons
- Need based on geography
- Need based on socioeconomic factors
- Need based on gravida
- Previous experience with birth in the facility

1. In your opinion, what are the reasons pregnant women **might use or not use a mother’s shelter**? (PROBE FOR: social, cultural, economic structural factors. Your questions should explore the following types of reasons:

**PROBE FOR**:

- Social/cultural reasons
- Financial implications
- Perceptions of health and personal safety
- Permission/support from family
- Encouragement or discouragement from peers

1. What kind of **support from families or to families** would pregnant women need to obtain before using the mother’s shelter?

**PROBE FOR**:

- Whose support/permission would be needed
- Help with childcare/housekeeping during the absence
- Help with transport
- Help with costs
- Person to accompany

1. Which persons should be allowed to stay with pregnant women in the mother’s shelter?

**PROBE FOR**:

- Children, including number and age
- Other family members
- Traditional birth attendants
- Other support persons

1. What would be the essential things (elements) that would make a mother’s shelter good for pregnant women and their families?

**PROBE FOR**:

- Aspects of the structure
- Amenities, furnishings, supplies
- Issues related to food/cooking
- Types of staff on hand to assistant women
- Other factors

1. What can the community do to support the mother’s shelters?
2. Would you be willing to pay to stay in a mother’s shelter? If so, how much – in money or in-kind donation?
3. Do you have any other thoughts to share about this subject?
