# Supplementary material for: Experiences with and expectations of maternity waiting homes in Luapula Province, Zambia: a mixed–methods, cross-sectional study with women, community groups and stakeholders
Source: BMC Pregnancy Childbirth. 2018 Jan 25;18:42. doi: 10.1186/s12884-017-1649-1 (PMC5785796; doi:10.1186/s12884-017-1649-1)
Supplement: Supplementary file 2 — Maternity Home Assessment Tool – document used to collect data about the structure and amenities available at each maternity home. (DOC 219 kb) [file 12884_2017_1649_MOESM2_ESM.doc]

**MATERNITY HOME ASSESSMENT TOOL**

|  | **District Code:** _____________________  **Facility ID Code**:___________________ |  |
| --- | --- | --- |
|  | **Interview’s ID code**:_____________  **Date**______________________________ |  |
| 1 | Locality of facility: |  |
|  | Rural | 1 |
|  | Peri-urban | 2 |
|  | Urban | 3 |
| 2 | Which group is the owner of the mother’s shelter? |  |
|  | Ministry of Health/Government | 1 |
|  | Mission | 2 |
|  | Community | 3 |
|  | Private | 4 |
|  | Other specify____________________________ | 5 |
| 3 | Which of the following groups contribute funds to the functioning of the mother’s shelter? (CHECK ALL THAT APPLY) |  |
|  | Ministry of Health/Government | 1 |
|  | Private funds | 2 |
|  | Charity/Mission Hospital | 3 |
|  | Income-generating activities | 4 |
|  | Self-paying clients | 5 |
|  | In-kind community contribution | 6 |
|  | Other specify:______________________________________ | 7 |

| 7 | What’s the main material used for the mother’s shelter’s walls? |  |
| --- | --- | --- |
|  | Cane/palm/trunks | 1 |
|  | Mud | 2 |
|  | Bamboo/pole with mud | 3 |
|  | Stone with mud | 4 |
|  | Plywood | 5 |
|  | Cardboard | 6 |
|  | Reused wood | 7 |
|  | Cement | 8 |
|  | Stone with lime/cement | 9 |
|  | Bricks | 10 |
|  | Cement blocks | 11 |
|  | Wood planks/shingles | 12 |
|  | Other/missing | 13 |
| 8 | How many bedrooms are present in the mother’s shelter? |  |
| 9 | How many beds are available for pregnant or postpartum women in the mother’s shelter? |  |
| 10 | How many beds are available for pregnant or postpartum women’s companions? |  |
| 11 | How many toilet facilities are available in the facility? |  |
| 12 | What kind of toilet facilities? (CHECK ALL THAT APPLY) |  |
|  | Flush/pour flush to piped sewer system | 1 |
|  | Flush/pour to septic tank | 2 |
|  | Ventilated improved pit latrine | 3 |
|  | Pit latrine with slab | 4 |
|  | Composting toilet | 5 |
|  | Any facility shared with other facilities | 6 |
| 13 | What is the source of electricity for this facility? |  |
|  | National grid (ZESCO) | 1 |
|  | Generator | 2 |
|  | Solar | 3 |
|  | None | 4 |
|  | Other specify:___________ | 5 |
| 14 | Does this facility have electricity today? |  |
|  | Yes | 1 |
|  | No | 2 |

| 15 | What is the main source of the water used in the MWH today? |  |
| --- | --- | --- |
|  | Piped water into dwelling/yard/plot | 1 |
|  | Public tap/standpipe | 2 |
|  | Protected dug well | 3 |
|  | Unprotected dug well | 4 |
|  | Tanker truck/cart with small tank | 5 |
|  | Surface water | 6 |
|  | Bottled water, improved source for cooking/washing | 7 |
|  | Other | 8 |
| 16 | How is water treated prior to drinking? |  |
|  | Boiled | 1 |
|  | Clorin | 2 |
|  | Strained through cloth | 3 |
|  | Ceramic, sand or other filter | 4 |
|  | Solar disinfection | 5 |
|  | Other | 6 |
|  | No treatment | 7 |
| 17 | How is water stored before use? |  |
|  | Not stored | 1 |
|  | Plastic bucket with a lid /jerry can | 2 |
|  | Bucket without a lid /basin | 3 |
|  | Other (specify)___________ | 4 |
| 18 | What place for cooking is available in the mother’s shelter? |  |
|  | Separate kitchen | 1 |
|  | No separate kitchen | 2 |
|  | Separate building | 3 |
|  | Outdoors | 4 |
|  | Other (specify _______________) | 5 |
| 19 | Who is allowed to use the cooking facilities? (CHECK ALL THAT APPLY) |  |
|  | Hospital/health centre staff | 1 |
|  | Mother’s shelter staff | 2 |
|  | Pregnant/postpartum women staying at the mother’s shelter | 3 |
|  | Companions of pregnant/postpartum women | 4 |
|  | Other specify:__________________________ | 5 |

| 20 | What cooking fuel is available? | | | | |  | |
| --- | --- | --- | --- | --- | --- | --- | --- |
|  | Electricity | | | | | 1 | |
|  | LPG/natural gas/biogas | | | | | 2 | |
|  | Kerosene | | | | | 3 | |
|  | Coal/ignite | | | | | 4 | |
|  | Charcoal | | | | | 5 | |
|  | Wood | | | | | 6 | |
|  | Straw/shrubs/grass | | | | | 7 | |
|  | Agricultural crop | | | | | 8 | |
|  | None available | | | | | 9 | |
| 21 | Are there posters on nutrition, child care, family planning in the MWH? | | | | |  | |
|  | Yes pasted on the walls | | | | | 1 | |
|  | Yes, hidden in a cupboard | | | | | 2 | |
|  | No | | | | | 3 | |
|  | | Check the amenities that belong to the MWH and are available for pregnant/postpartum women staying at the facility to use | 22. Provided by the facility? | | 23. In good condition? | | |
|  | |  | **Yes** | **No** | **Yes** | | **No** |
| a. | | Beds | 1 | 2 | 1 | | 2 |
| b. | | Mattresses | 1 | 2 | 1 | | 2 |
| c. | | Linens | 1 | 2 | 1 | | 2 |
| d. | | Cooking pots | 1 | 2 | 1 | | 2 |
| e. | | Plates | 1 | 2 | 1 | | 2 |
| f. | | Spoons | 1 | 2 | 1 | | 2 |
| g. | | Cooking utensils | 1 | 2 | 1 | | 2 |
| h. | | Bed nets | 1 | 2 | 1 | | 2 |
| i. | | Telephone | 1 | 2 | 1 | | 2 |
|  | |  | | | | |  |
| 24. | | Do pregnant/postpartum women staying at the mother’s shelter receive health talks/education? | | | | |  |
|  | | Yes | | | | | 1 |
|  | | No | | | | | 2 |
| 25. | | What topics are covered in the educational sessions? | | | | |  |
|  | | Maternal/neonatal nutrition | | | | | 1 |
|  | | Family planning | | | | | 2 |
|  | | Neonatal care/breastfeeding | | | | | 3 |
|  | | Child care | | | | | 4 |
|  | | Other | | | | | 5 |

| 26. | Which of the following food is regularly available to mothers? (CIRCLE THE BEST RESPONSE) | | | |
| --- | --- | --- | --- | --- |
|  |  | **Yes**, MWH offers supplies of this food | **Yes**, women bring to MWH | **No**, not available |
| 26a. | Body building (foods (Meat/fish/poultry/eggs, milk products, groundnuts, beans) | 1 | 2 | 3 |
| 26b. | Energy foods (maize, sorghum, millet, cassava, sweet potatoes, Irish potatoes, bread/scones) | 1 | 2 | 3 |
| 26c. | Protective foods (Vitamin-A rich fruits/vegetables,) | 1 | 2 | 3 |
|  |  |  |  |  |
| **Liquids** | | | | |
| 26d. | Milk | 1 | 2 | 3 |
| 26e. | Tea/Coffee | 1 | 2 | 3 |
| 26f. | Other Liquids | 1 | 2 | 3 |

| 27. | | Have facility staff advocated to traditional leaders, decision makers, NHCs and SMAGs on the importance of MWHs in the past year? INTERVIEWER: read each topic and circle correct response. | | | | | | | |
| --- | --- | --- | --- | --- | --- | --- | --- | --- | --- |
|  | |  | **Yes** | **No** | | | **Don’t Know** | | |
| 27a. | | Safe pregnancy, labor and delivery | 1 | 2 | | | 9 | | |
| 27b. | | Birth planning, emergency preparedness in  Pregnancy | 1 | 2 | | | 9 | | |
| 27c. | | Recognition of danger signs in pregnancy,  labor; prolonged labor | 1 | 2 | | | 9 | | |
| 27d. | | Harmful traditional practices | 1 | 2 | | | 9 | | |
| 27e. | | Family planning | 1 | 2 | | | 9 | | |
| 27f. | | Gender issues, gender relations, gender equity | 1 | 2 | | | 9 | | |
| 27g. | | Other (specify):_____________________ | 1 | 2 | | | 9 | | |
| 28 | Who cleans the MWH at this facility? (CHECK ALL THAT APPLY) | | | | | | |  | |
|  | Volunteer | | | | | | | 1 | |
|  | Employee paid by the organization | | | | | | | 2 | |
|  | Employee paid by the community | | | | | | | 3 | |
|  | Pregnant women | | | | | | | 4 | |
|  | Accompanying relatives | | | | | | | 5 | |
|  | NHC/SMAGs members | | | | | | | 6 | |
| 29 | How often is routine maintenance of the MWH done? | | | | | | |  | |
|  | Never | | | | | | | 1 | |
|  | Every six months | | | | | | | 2 | |
|  | Every year | | | | | | | 3 | |
|  | Whenever necessary | | | | | | | 4 | |
| 30 | Does this facility have a formal system for reviewing management or administrative issues related to the mother’s shelter (maternity waiting home)? | | | | | | |  | |
|  | Yes | | | | | | | 1 | |
|  | No | | | | | | | 2 | |
|  | Don’t know | | | | | | | 8 | |
| 31 | How often are formal meetings & discussions held to discuss the facility’s management or administrative issues? | | | | | | |  | |
|  | Weekly | | | | | | | 1 | |
|  | Monthly | | | | | | | 2 | |
|  | Quarterly | | | | | | | 3 | |
|  | Semiannually | | | | | | | 4 | |
|  | Don’t know | | | | | | | 8 | |
| 32 | When was the last such meeting held? (DD/MM/YY) | | | | | | | __/__/ __ | |
|  |  | | | | Yes | No | | | Don’t  know |
| 33 | Are there any formal tools or approaches used for quality improvement activities? | | | | 1 | 2 | | | 8 |
| 34 | Is there a system in place to determine client opinion about the MWH services? | | | | 1 | 2 | | | 8 |
| 35 | Does the facility have written protocols/guidelines for infection prevention in the MWH or for the disposal of contaminated items? | | | | 1 | 2 | | | 8 |

| 36 | List the types of record keeping for MWH? CHECK ALL THAT APPLY. | | | | | |
| --- | --- | --- | --- | --- | --- | --- |
|  | | General state | Completeness | Confidentiality | Adequacy for indicators |  |
| a. Client record | |  |  |  |  |  |
| b. Admission record | |  |  |  |  |  |
| c. Discharge register | |  |  |  |  |  |
| d. Other (specify) | |  |  |  |  |  |
| If mothers’ shelter registers are unavailable, ask the respondent who answered the previous question to answer the following questions. , | | | | | | |
| 37 | How many pregnant women are staying at the mother’s shelter today? | | | | |  |
| 38 | How many persons accompanying pregnant women are staying at the mother’s shelter today? | | | | |  |
| 39 | How many postpartum women are staying at the mother’s shelter today? | | | | |  |
| 40 | How many persons accompanying postpartum women are staying at the mother’s shelter today? | | | | |  |
| 41 | How many pregnant or postpartum women have stayed at the mother’s shelter the past three months? | | | | |  |
| 42 | How many companions of pregnant or postpartum women have stayed at the mother’s shelter during that past three months? | | | | |  |

**THANK YOU FOR YOUR TIME**
