# Supplementary material for: Experiences with and expectations of maternity waiting homes in Luapula Province, Zambia: a mixed–methods, cross-sectional study with women, community groups and stakeholders
Source: BMC Pregnancy Childbirth. 2018 Jan 25;18:42. doi: 10.1186/s12884-017-1649-1 (PMC5785796; doi:10.1186/s12884-017-1649-1)
Supplement: Supplementary file 3 — Service Abstraction Form – document used to extract data on deliveries from maternity home registers. (DOC 31 kb) [file 12884_2017_1649_MOESM3_ESM.doc]

**SERVICE ABSTRACTION FORM (IN PART)**

**Instruction to the Interviewer:** Review the waiting homes registers, count the numbers and enter the totals for the items in the table below.

| **Maternity Waiting Home (MWH) Register** | |
| --- | --- |
| Number of women who stayed in the MWH |  |
| Number of women present in MWH |  |
| Number of companions |  |
| Average duration of stay |  |
